# Knowledge of SARS-CoV-2 antigen detection and proper use of rapid diagnostic self-test among Shanghai residents in China

**DOI:** 10.3389/fpubh.2023.1036823

**Published:** 2023-01-24

**Authors:** Ren-Ping Gu, Ai-Yong Zhu

**Affiliations:** School of Nursing and Health Management, Shanghai University of Medicine and Health Sciences, Shanghai, China

**Keywords:** knowledge, SARS-CoV-2, self-test, COVID-19, antigen-detection

## Abstract

**Objectives:**

To assess and evaluate the knowledge of Shanghai, China, residents on the use of SARS-CoV-2 antigen detection and rapid diagnostic self-test.

**Methods:**

A cross-sectional electronic survey using a self-administered questionnaire was sent *via* the online platform, Sojump, to general individuals. Multiple linear regression analysis was performed to determine the variables associated with knowledge of self-test.

**Results:**

A total of 283 participants were recruited between July 1, 2022 and July 20, 2022 through an online survey. The mean score of knowledge on the tests was 14.33 ± 2.85 (out of 21). The questions concerning the depth of swab insertion and minimum number of swab rotations in the nostril, necessity of bilateral sampling, necessity of rotating and squeezing the swab for 10 times in the extraction buffer tube, and waiting time for the results showed the highest rate of incorrect responses. In the multiple regression analysis model, sex, social status, and source of information were associated with the knowledge on the self-test kits.

**Conclusion:**

Immediate health education programs should be made available and the kits could be improved appropriately to ensure adequate knowledge. The use of technology should be fully leveraged to achieve accurate self-diagnosis and correct interpretation of the results.

## Introduction

On March 1, 2022, the Shanghai Municipal Health Commission reported new cases of symptomatic and asymptomatic patients with Coronavirus disease (COVID-19) (1), marking the beginning of a new wave of COVID-19 outbreaks in Shanghai. Diagnostic testing for severe acute respiratory syndrome coronavirus 2 (SARS-CoV-2) is a key component to control the current COVID-19 pandemic (2). To further optimize the detection strategy of the novel coronavirus and meet the needs of pandemic prevention and control, the State Council's Integrated Group for Joint Prevention and Control of the Novel Coronavirus Pneumonia decided to include antigen tests as a supplement to nucleic acid tests, which led to the formulation of the “Antigen Test Scheme for the Novel Coronavirus (Trial)” (3). The scheme specifies four types of scenarios/places where antigen testing can be used: (1) grassroots medical and health service institutions; (2) isolation and quarantine facilities; (3) community self-testing of (village) residents; and (4) other individuals for whom monitoring should be strengthened.

Antigen-detection rapid diagnostic tests (Ag-RDTs) for SARS-CoV-2 provide immediate results without the need for a laboratory; and are easy and economical to use (2, 4). The user-friendly characteristics of Ag-RDTs enable them to be considered for self-testing. The World Health Organization recommended that an Ag-RDT test must have a sensitivity of at least 80% and a specificity of at least 97%, based on the gold-standard real-time reverse transcriptase-polymerase chain reaction (RT-PCR) test (2). Current evidence showed a reasonably good sensitivity (82.5–97%) and high specificity (99.1–100%) of COVID-19 self-testing to identify individuals with a high probability of contagiousness (5–7). A French study showed that the reliability between the self-test Ag-RDT and the multiplex molecular detection of SARS-CoV-2 RNA was 0.94 (8). Besides, the self-collection procedure could be performed easily by most participants (5, 9). Antigen testing in Shanghai during the outbreak was performed in the form of self-tests with nasal mid-turbinate (NMT) sampling. Citizens underwent received SARS-CoV-2 antigen rapid test kits for free (components including swab, extraction buffer tube, test cassette, sample bag, and instruction leaflet), and were requested to collect the specimen on their own and report the corresponding results to health authorities. A positive antigen test result must be reported before performing a confirmatory nucleic acid test during which the suspected patient will be isolated to avoid further transmission. As of April 29, 2022, the State Food and Drug Administration approved 31 antigen test kits for the novel coronavirus (10). Ag-RDTs are easier to perform than nucleic acid amplification tests (NAATs); however, the disadvantages need careful consideration. With the low sensitivity of RDTs to RT-PCR, RT-PCR is considered the gold standard for the detection of SARS-CoV-2 (11). Deceitful methods may easily lead to misuse of COVID-19 Ag-RDTs and lead to false-positive results (12). Unreasonable sampling will lead to false-negative results, and strict compliance to manufacturer-recommended procedures is still necessary (2).

This study aimed to assess and evaluate the knowledge of Shanghai residents on SARS-CoV-2 antigen detection and proper use of rapid diagnostic self-test.

## Methods

### Data collection

Convenience sampling was used to recruit adult participants by means of an online questionnaire between July 1 and July 20, 2022. At this time, antigen self-test was still an important complementary means for quarantined people, special occupational groups, medical institutions, and residents needing self-testing. Participants were recruited *via* the Sojump website (https://www.wjx.cn/), which is the most commonly used online survey tool in China. Inclusion criteria were ≥18 years of age and had tested with SARS-CoV-2 antigen detection and rapid diagnostic self-test in the past 3 months. Only one respondent per questionnaire was permitted. We determined the sample size using the Raosoft sample size calculator (http://www.raosoft.com/samplesize.html). The required sample size was 246 with a 5% margin of error and a confidence interval of 95% based on the population of 22,000,000 and a response distribution of 80%.

### Questionnaire design

The anonymous online questionnaire was designed based on the interim guidance of the World Health Organization for antigen detection in the diagnosis of SARS-CoV-2 infection (2) and the manufacturer's instructions for use (13). The questionnaire was divided into three sections. The first section included the demographic characteristics of the residents: sex, age, education, marital status, region, social status, and monthly family income. Section 2 included 21 questions on knowledge on SARS-CoV-2 antigen detection and rapid diagnostic self-testing; six questions on sample collection, four on test performance, three on result interpretation, four on test kit storage, two on waste management, and two on reporting of results. Section 3 included one question on the source of knowledge of first-time use of antigen self-test kits. Respondents were requested to select the answers they believe were correct based on their own knowledge. Regarding questions K11, K12, and K13, all the correct answers were selected. Incomplete answers were considered as partially correct answers. To minimize the possibility of participants selecting a random answer, the choice of “I Don't Know” was added to some questions (Supplementary Table). Each question was scored 1 point for correct answers, 0.5 for partially correct answers in multiple response questions, and 0 for incorrect or unknown answers. The sum of the individual scores for all questions was the total score (range, 0–21), with higher scores indicating better knowledge of SARS-CoV-2 antigen detection and rapid diagnostic self-test. Participants read an informed consent statement before starting the survey. By clicking on a “yes” button, participants were informed that they were providing consent to complete the survey. A pilot study was conducted in 20 college students on June 27, 2022, to ensure that the questionnaire could be widely accepted and fully understood. All the procedures performed in this study were in accordance with the 1964 Helsinki Declaration and its later amendments. This study was approved by the Institutional Ethics Review Board of Shanghai University of Medicine & Health Sciences (2022-zxkt-02-310225198301136663).

### Statistical analysis

We used SPSS V.21.0 for statistical analysis. Demographic characteristics and knowledge of SARS-CoV-2 antigen detection and rapid diagnostic self-tests were measured using descriptive statistics. *T*-test or one-way ANOVA test were performed to determine the significant relations of the mean knowledge scores with socio-demographic information. The variables found by the univariate analysis as significantly associated with the outcomes were considered for the multivariate analyses. The level of statistical significance was 0.05.

## Results

A total of 288 participants responded to the questionnaire. Five participants were excluded because they were under 18 years of age. The mean (SD) age of the participants was 34.87 (11.47) years, among whom 117 (41.3%) were men. Most participants had a college degree (84.4%); 171 (60.4%) were single; and 258 (91.2%) did not live alone. The majority (84.1%) of the participants lived in urban areas. There were 176 (62.2%) participants who had a monthly family income of 10,000–50,000 yuan (1,400–7,000$). The dominant source of knowledge regarding the first time use of self-test kits was video (60.4%), followed by instruction manuals (33.6%), guidance from relatives and friends (4.9%), and others (1.1%).

The highest possible score was 21, and the average knowledge score of the study participants was 14.33 ± 2.85 with an overall correct answer rate of 68% (14.33/21^*^100). Overall, female participants had better knowledge than male participants (*p* < 0.05). There was a significant difference in total knowledge scores between the sources of self-test knowledge for the first-time user group. The univariate analysis results showed that the scores of result interpretation, kit storage, waste management, and reporting of results were significantly different between age groups. Marital status was also recognized to have a significant association with scores on reporting of results (*p* < 0.05). Likewise, it was found that those not living alone had greater scores on reporting of results than those living alone (*p* < 0.05) (Table 1).

**Table 1:** Association between demographic variables and knowledge of self-tests (M ± SD).

**Variables**	***N* (%)**	**Total score (range 0–21)**	**Sample collection (range 0–6)**	**Test performance (range 0–4)**	**Results interpretation (range 0–3)**	**Kit storage (range 0–4)**	**Waste management (range 0–2)**	**Results reports (range 0–2)**
Overall	283 (100.0)	14.33 ± 2.85	3.47 ± 1.20	2.24 ± 0.74	2.09 ± 0.82	3.05 ± 1.00	1.83 ± 0.43	1.64 ± 0.58
**Sex**								
Male	117 (41.3)	13.60 ± 3.15	3.24 ± 1.14	2.10 ± 0.82	1.95 ± 0.86	2.88 ± 1.12	1.82 ± 0.48	1.61 ± 0.66
Female	166 (58.7)	14.84 ± 2.51	3.63 ± 1.21	2.34 ± 0.67	2.19 ± 0.79	3.16 ± 0.88	1.84 ± 0.40	1.67 ± 0.52
*P*		0.001^**^	0.006^**^	0.009^**^	0.016^*^	0.024^*^	0.664	0.397
**Age (years)**								
18–29	101 (35.7)	13.92 ± 3.28.	3.58 ± 1.20	2.26 ± 0.73	2.04 ± 0.92	2.85 ± 1.02	1.73 ± 0.55	1.46 ± 0.70
30–39	63 (22.3)	14.60 ± 2.62	3.48 ± 1.16	2.14 ± 0.86	2.26 ± 0.73	3.06 ± 0.97	1.94 ± 0.25	1.71 ± 0.49
40–49	103 (36.4)	14.60 ± 2.46	3.33 ± 1.17	2.25 ± 0.65	2.08 ± 0.77	3.26 ± 0.96	1.90 ± 0.33	1.77 ± 0.47
50–59	7 (2.5)	15.36 ± 4.23	3.86 ± 1.87	2.43 ± 1.27	2.50 ± 0.76	3.00 ± 1.15.	1.57 ± 0.79	2.00 ± 0.00
≥60	9 (3.2)	13.11 ± 1.39	3.44 ± 1.24	2.44 ± 0.53	1.33 ± 0.43	2.67 ± 0.87	1.67 ± 0.50	1.56 ± 0.53
*P*		0.193	0.550	0.685	0.014^*^	0.039^*^	0.003^**^	0.001^**^
**Education**								
≤ High school	44 (15.5)	13.98 ± 2.75	3.20 ± 1.34	2.32 ± 0.56	1.91 ± 0.82	3.00 ± 0.99	1.84 ± 0.43	1.70 ± 0.59
≥Some college	239 (84.4)	14.39 ± 2.87	3.52 ± 1.17	2.23 ± 0.77	2.13 ± 0.82	3.05 ± 1.00	1.83 ± 0.43	1.63 ± 0.58
*P*		0.377	0.110	0.450	0.106	0.740	0.908	0.446
**Marital status**								
Married	171 (60.4)	14.40 ± 2.65	3.35 ± 1.13	2.22 ± 0.77	2.11 ± 0.77	3.14 ± 0.98	1.86 ± 0.40	1.74 ± 0.49
Single	100 (35.3)	14.20 ± 3.06	3.62 ± 1.20	2.29 ± 0.70	2.10 ± 0.90	2.92 ± 1.00	1.77 ± 0.51	1.50 ± 0.67
Divorced/widowed	10 (3.5)	14.60 ± 3.68	4.10 ± 1.60	2.10 ± 0.74	1.90 ± 0.97	2.90 ± 1.10	2.00 ± 0.00	1.60 ± 0.70
Remarried	2 (0.7)	12.75 ± 6.72	3.50 ± 3.53	2.50 ± 0.71	1.75 ± 1.06	2.00 ± 1.41	2.00 ± 0.00	1.00 ± 0.00
*P*		0.797	0.102	0.749	0.818	0.136	0.211	0.004^**^
**Region**								
Urban	238 (84.1)	14.43 ± 2.79	3.50 ± 1.21	2.26 ± 0.75	2.11 ± 0.80	3.08 ± 0.97	1.85 ± 0.42	1.65 ± 0.57
Rural	45 (15.9)	13.78 ± 3.12	3.31 ± 1.13	2.16 ± 0.74	2.02 ± 0.97	2.89 ± 1.15	1.78 ± 0.52	1.62 ± 0.65
*P*		0.159	0.333	0.405	0.527	0.250	0.345	0.793
**Social status**								
Living alone	25 (8.8)	12.74 ± 4.17	3.08 ± 1.15	2.20 ± 0.87	1.90 ± 1.08	2.64 ± 1.29	1.64 ± 0.70	1.28 ± 0.94
Living with others	258 (91.2)	14.48 ± 2.65	3.51 ± 1.20	2.24 ± 0.73	2.11 ± 0.80	3.09 ± 0.96	1.85 ± 0.40	1.68 ± 0.52
*P*		0.051	0.088	0.777	0.347	0.104	0.147	0.046^*^
**Monthly family income**								
Under 10,000 yuan (1,400$)	53 (18.7)	14.13 ± 2.68	3.58 ± 1.22	2.28 ± 0.60	1.85 ± 0.82	2.91 ± 1.01	1.83 ± 0.38	1.68 ± 0.55
10,001–20,000 yuan (1,401–2,800$)	90 (31.8)	14.36 ± 2.76	3.47 ± 1.19	2.21 ± 0.68	2.17 ± 0.84	3.09 ± 0.92	1.86 ± 0.41	1.57 ± 0.64
20,001–50,000 yuan (2,801–7,000$)	86 (30.4)	14.62 ± 2.79	3.45 ± 1.14	2.28 ± 0.76	2.19 ± 0.79	3.19 ± 1.02	1.83 ± 0.47	1.69 ± 0.54
Above 50,000 yuan (7,000$)	54 (19.1)	14.00 ± 3.26	3.39 ± 1.30	2.19 ± 0.93	2.06 ± 0.83	2.89 ± 1.06	1.82 ± 0.48	1.67 ± 0.58
*P*		0.598	0.862	0.840	0.081	0.236	0.948	0.509
**Source of self-test knowledge for the first time**								
Video	171 (60.4)	14.60 ± 2.61	3.53 ± 1.15	2.29 ± 0.72	2.06 ± 0.88	3.12 ± 0.90	1.88 ± 0.36	1.71 ± 0.53
Manufacturer instructions	95 (33.6)	14.23 ± 3.09	3.49 ± 1.26	2.23 ± 0.78	2.21 ± 0.71	2.96 ± 1.08	1.81 ± 0.49	1.53 ± 0.65
Relatives and friends	14 (4.9)	12.68 ± 2.60	2.64 ± 1.08	2.00 ± 0.56	1.68 ± 0.77	3.07 ± 1.00	1.50 ± 0.65	1.79 ± 0.43
Others	3 (1.1)	9.67 ± 3.79	3.00 ± 1.00	1.00 ± 0.00	2.00 ± 1.00	1.33 ± 2.31	1.33 ± 0.58	1.00 ± 1.00
*P*		0.002^**^	0.053	0.014^*^	0.138	0.013^*^	0.002^**^	0.014^*^

M, median; SD, standard deviation.

^*^p < 0.05, ^**^p < 0.01.

Higher knowledge score indicates better knowledge.

Knowledge of SARS-CoV-2 antigen detection and rapid diagnostic self-test, Shanghai, China, 2022.

**Figure 1:**
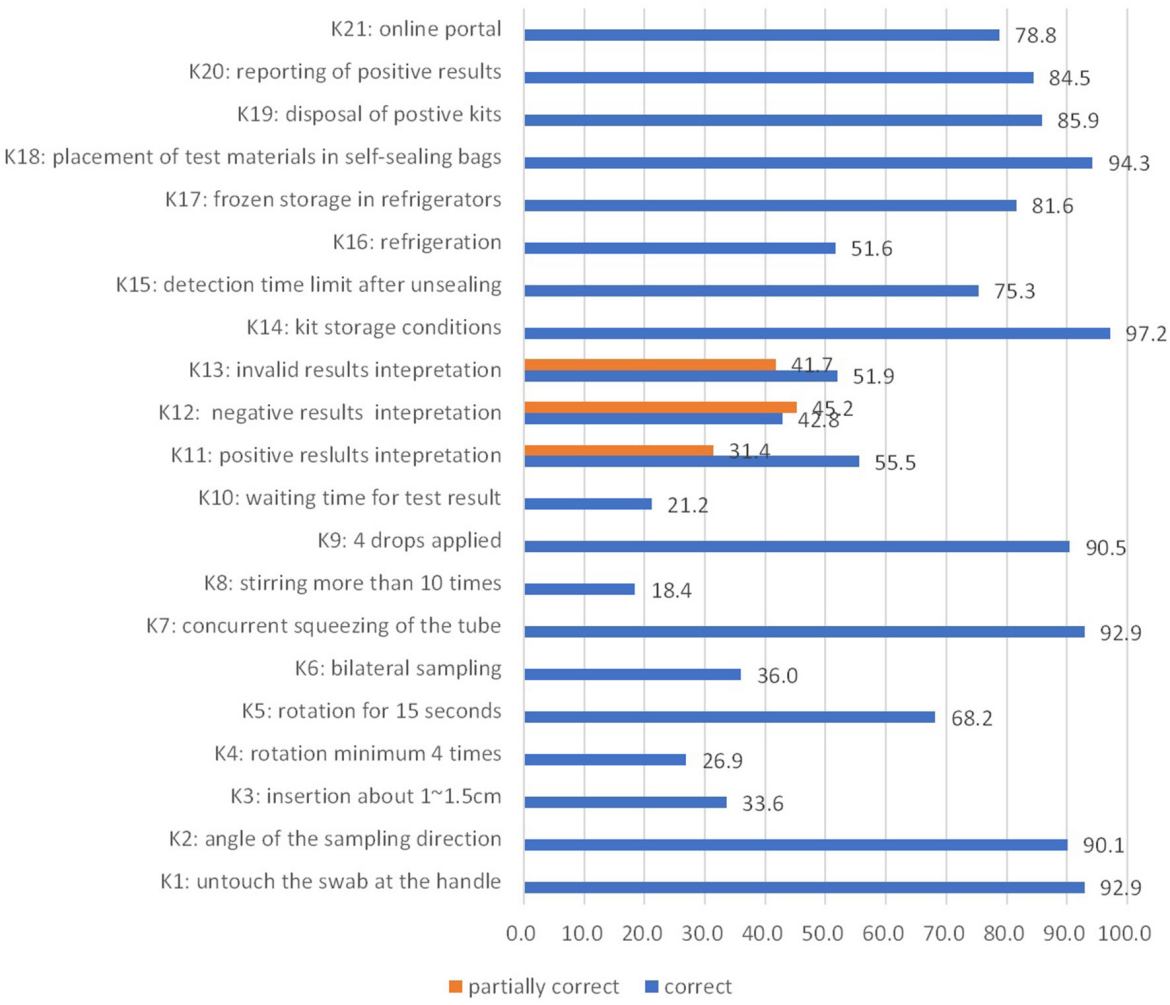
Knowledge score (%) in the study (knowledge of SARS-CoV-2 antigen detection and rapid diagnostic self-test, Shanghai, China, 2022).

The questions concerning the depth of swab insertion (~1–1.5 cm) and minimum number of swab rotations (at least four times) into the nostril, necessity of bilateral sampling, necessity of rotating and squeezing the swab for 10 times in the extraction buffer tube, and waiting time for the results showed the highest rate of incorrect responses (Figure 1).

In the association between demographic variables and the scores on knowledge of the participants using the multivariate linear regression analysis, a significant association was found regarding sex, social status, and source of knowledge on SARS-CoV-2 antigen detection and rapid diagnostic self-test (Table 2). Female sex and age were associated with increased knowledge (*p* < 0.05). Source of knowledge was associated with less positive increase in knowledge (*p* < 0.05).

**Table 2:** Multivariate linear regression analysis on the knowledge related to SARS-CoV-2 antigen-detection rapid diagnostic self-tests.

**Variables**	**B**	**SE**	**β**	***t***	***p***
Sex	1.05	0.34	0.18	3.09	0.002^**^
Age	0.29	0.19	0.11	1.54	0.126
Marital status	0.29	0.34	0.06	0.85	0.398
Social status	1.68	0.62	0.17	2.72	0.007^**^
Source of self-test knowledge for the first time	−0.79	0.26	−0.18	−3.10	0.002^**^

B, unstandardized regression coefficient; SE, standard error; β, standardized regression coefficient.

^**^p < 0.01.

Knowledge of SARS-CoV-2 antigen detection and rapid diagnostic self-test, Shanghai, China, 2022.

## Discussion

Symptom based diagnosis and contact tracing of close contacts alone are insufficient in preventing the ongoing pandemic of COVID-19 (14). Rapid and early detection is essential for controlling the COVID-19 pandemic (2). Real-time reverse transcriptase-polymerase chain reaction (RT-PCR) is considered the gold standard for the detection of SARS-CoV-2 (11). However, diagnosis with SARS-CoV-2 RT-PCR is not suitable for mass use due to the nature of its long processing time and a need for specialized laboratories and trained personnel. Moreover, RT-PCR tests can cause delays in reporting positive results, eventually leading to delays in contact tracing (4). Ag-RDTs can complement the diagnosis of large populations owing to the rapidity, inexpensiveness, sufficient sensitivity, and ease of specimen self-collection of the kits (4). During this wave of the pandemic, residents in Shanghai perform antigen kits by themselves before RT-PCR tests. If the antigen test is positive, it suggests a greater possibility of infection. They do not go out for centralized RT-PCR tests because all residents go to a designated place for RT-PCR tests at the same time. Professional personnel collect these RT-PCR test samples separately and confirm whether they are infected, which can avoid the risk of cross infection caused by centralized RT-PCR tests (15). In this process, antigen tests, as a preliminary screening method, is effectively complementary to RT-PCR tests. Although the accuracy of antigen tests self-administered by a lay person is comparable to that performed by professionals (16), effective management is still needed to ensure the accuracy of test results and to achieve timely surveillance of the pandemic by the public health system.

Our study revealed that among participants from Shanghai, China, there was a moderate level of knowledge regarding the SARS-CoV-2 antigen detection and rapid diagnostic self-test. The level of knowledge reflected by the correct answer scores (68%) in the present study is poorer than that in a French study, wherein 94.4% of the participants correctly answered all 11 questions on comprehension of labeling after reading or watching the manufacturers' instruction and declaring full comprehension (8). In our study, more specific questions were included besides those on sample collection, test performance, and result interpretation.

The results showed that female participants reported better knowledge of SARS-CoV-2 antigen detection and rapid diagnostic self-tests than the male participants. Surveys on novel coronavirus knowledge also reveal that women are more knowledgeable than men (17, 18). One reason may be that females are generally more concerned about seeking and practicing health information than males (17).

The National Health Commission has produced official videos that can be viewed on the Internet, television, and WeChat (the most popular social media platform in China). Overall, participants showed better knowledge of Ag-RDTs for SARS-CoV-2 when using audiovisual or manufacturer instructions compared to when the source of information came from other people or word of mouth. Official video and manufacturer instructions are reliable sources of information in learning important knowledge about pandemic prevention and control. Compared with other sources of information, official video is more popular among the general public (5).

Participants who lived with their families had better knowledge than those who did not. This may be because in families with more members, there is more discussion, more exchange of knowledge, and more younger family members who can help the older ones to master new methods and techniques.

Our study results indicated that greater knowledge is not significantly associated with level of education. Similarly, in a cross sectional study in Cantabria, Spain, incorrect use of the self-collection rapid antibody test was not associated with the educational level (19). It indicated that understanding the instructions for self test was not the challenge for low educational level.

In this study, we found that participants had a poor knowledge on sample collection and test performance, with a correct answer rate of 57.8% (3.47/6^*^100) and 56.0% (2.24/4^*^100), respectively. Similarly, in a study on the feasibility of self-testing involving 146 adults with a high suspicion of SARS-CoV-2 infection, incorrect depth of insertion of the swab, reduced intensity of swabbing, unilateral nasal mid-turbinate sampling, and specimen extraction were the main deviations (5). Incorrect sampling will lead to false-negative results and immediate health education programs should be distributed to ensure adequate knowledge. Furthermore, the SARS-CoV-2 antigen rapid test kit could be improved appropriately. For example, a collar was added at 5.5 cm of the swab as a guide to the maximum insertion depth (20). Moreover, Ag-RDTs can be performed using self-collected nasal swab or saliva. A meta-analysis found that saliva had a similar diagnostic performance with that of nasopharyngeal and throat swabs (21). Saliva sampling has some advantages over nasal swab collection, including less discomfort and nasal bleeding. Additionally, information platforms have the potential to provide auxiliary support by allowing individuals to not only connect with the information departments of regulatory authorities through mobile devices such as smartphones, but also live streaming of the sampling/inspection process rather than just the inspection results to achieve traceability management of self-test results and provide the public with convenient and professional consultation channels. On April 16, 2022, “Epidemic Test”, a mobile app, was launched by the Chinese Academy of Information and Communications to assist individuals and institutions to conduct COVID-19 antigen tests in Shanghai. Individuals can report and check Ag-RDT results voluntarily at any time. However, in this wave of the pandemic, RT-PCR test is still the only recognized “pass certificate” in China. Though antigen screening has been widely used, the detection results are not connected to the pandemic prevention and control system platform in various regions. In future, mobile applications can be used to obtain data of the kits' manufacturers and make the detection results traceable. They can systematically support relevant departments to make decisions on the pandemic prevention and control and epidemic surveillance.

This study enhances our understanding of the performance of SARS-CoV-2 antigen detection and rapid diagnostic self-test. However, we cannot eliminate selection bias because of convenient sampling. Our study participants were predominantly those with a college level of education and those living in urban areas; these limitations should be considered when interpreting the findings. Further research should use more robust methodologies to investigate this topic.

## Data availability statement

The raw data supporting the conclusions of this article will be made available by the authors, without undue reservation.

## Ethics statement

The studies involving human participants were reviewed and approved by the Institutional Ethics Review Board of Shanghai University of Medicine and Health Sciences (2022-zxkt-02-310225198301136663). The patients/participants provided their written informed consent to participate in this study.

## Author contributions

R-PG was a major contributor in writing the manuscript. A-YZ analyzed and interpreted the data. All authors read and approved the final manuscript.
