# Supplementary material for: Knowledge of SARS-CoV-2 antigen detection and proper use of rapid diagnostic self-test among Shanghai residents in China
Source: Front Public Health. 2023 Jan 24;11:1036823. doi: 10.3389/fpubh.2023.1036823 (PMC9902921; doi:10.3389/fpubh.2023.1036823)
Supplement: Supplementary file 1 [file Table_1.DOCX]

SUPPLEMENTARY TABLE Knowledge of SARS-CoV-2 Ag-RDTs Self-test Questionnaire

| **Knowledge of SARS-CoV-2 Ag-RDTs Self-test** | **Options** |
| --- | --- |
| K1: you can touch the swab at the tip with the cotton | •Yes •No •I don‘t know |
| K2: tilt your head back slightly | •Yes •No •I don‘t know |
| K3: insert the swab about ( ) cm deep into the nostril | •0.5 cm •0.5-1 cm •1-1.5 cm •1.5-2 cm •I don‘t know |
| K4: rotate the swab for at least ( ) rotations | •1 rotation •2 rotations •3 rotations •4 rotations •I don‘t know |
| K5: the swab stays for ( ) seconds | •5 seconds •10 seconds •15 seconds •20 seconds •I don‘t know |
| K6: repeat the procedure with the same swab in the other nostril | •Yes •No •I don‘t know |
| K7: while squeezing the buffer tube in the lower part, stir the swab | •Yes •No •I don‘t know |
| K8: shake the tube ( ) times after pressing the nozzle cap tightly | •3 times •5 times •10 times •I don‘t know |
| K9: apply ( ) drops of the extracted specimen to the test device | •1 drop •2-4 drops •I don‘t know |
| K10: after dropping the sample, wait for the result to be valid within ( ) minutes | •10 minutes •20 minutes •30 minutes •I don‘t know |
| K11: positive results is/are^a^ | 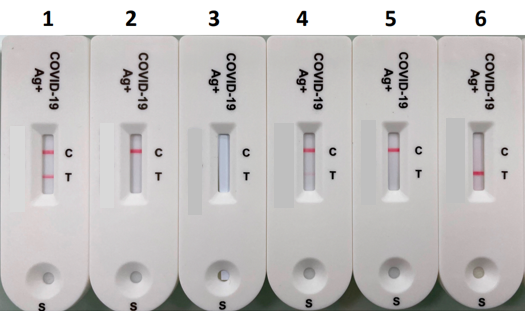  •1 •2 •3 •4 •5 •6 •I don‘t know |
| K12: negative results is/are^a^ |  |
| K13: invalid results is/are^a^ |  |
| K14: kit storage conditions | • in a cool place, away from bright light •in the sun light •I don‘t know |
| K15: detection time limit after unsealing | •1 hour •2 hours •3 hours •no time limit •I don‘t know |
| K16: the kits can be stored for refrigeration | •Yes •No •I don‘t know |
| K17: the kits can be frozen in refrigerators | •Yes •No •I don‘t know |
| K18: place the test materials in self-sealing bags | •Yes •No •I don‘t know |
| K19: how to dispose the positive kits^b^ |  |
| K20: what will you do with the positive results^b^ |  |
| K21: do you know the online portal | •Yes •No |

^a^ Respondents were requested to select all the correct answers. Incomplete answers were considered as partially correct answers.

^b^ These questions are open questions
